# Building models, building capacity: A review of participatory machine learning for HIV prevention

**DOI:** 10.1371/journal.pgph.0003862

**Published:** 2025-06-04

**Authors:** Mark Sendak, Meg Young, Jee Young Kim, Alifia Hasan, Clare Kelsey, Catherine O’Neal, Tonya Jagneaux, Wayne Wilbright, John Couk, Stephen Lim, Tamachia Davenport, Shirley Lolis, Jennifer Thomas, Shannon Widman, Suresh Balu, Meredith Clement, Lance Okeke

**Affiliations:** 1 Duke Institute for Health Innovation, Durham, North Carolina, United States of America; 2 Data and Society Research Institute, New York, New York, United States of America; 3 Louisiana State University Health Sciences Center New Orleans, New Orleans, Louisiana, United States of America; 4 Franciscan Missionaries of Our Lady Health System, Baton Rouge, Louisiana, United States of America; 5 LSU Health New Orleans, New Orleans, Louisiana, United States of America; 6 Camp ACE/ St John the Baptist #5, New Orleans, Louisiana, United States of America; 7 Metro Health, Baton Rouge, Louisiana, United States of America; 8 Duke University School of Medicine, Durham, North Carolina, United States of America; NYU Grossman School of Medicine: New York University School of Medicine, UNITED STATES OF AMERICA

## Abstract

A growing number of researchers and practitioners are embracing a “participatory turn” in machine learning (ML) to improve model development, prevent harm, and provide communities more influence over systems that impact them. In this paper, we explore the intersection of participatory practices in healthcare and the emerging focus on responsible AI with a focus on human immunodeficiency virus (HIV) care. We review the historical context of participation in HIV treatment and prevention, emphasizing how patient activism has shaped practices in this field. We then review participatory ML in HIV prevention and present a brief case study of a project designed to identify candidates for pre-exposure prophylaxis (PrEP) in Louisiana. The review highlights the essential steps in conducting participatory ML. Finally, we draw lessons for future participatory ML projects, underscoring the importance of long-term collaboration, responsiveness to partner feedback, and the crucial role of capacity-building for individuals and organizations. Effective participation requires substantial resources and investment, which supports overall project goals beyond mere improvements in model performance. We also draw lessons for advancing the participatory ML field, including (1) the impact of funding mandates on fostering effective engagement; (2) the need to scale participatory processes rather than just technologies; and (3) the need for genuine participation to allow flexibility in project plans, timelines, and shifts in institutional power dynamics.

## 1 Introduction

Researchers and practitioners are increasingly turning to impacted communities to co-develop AI systems and governance in what has been called a “participatory turn” in AI [1,2]. Drawing from participatory design principles in human-computer interaction [3], the participatory turn aims to help systems respond to the needs and concerns of people most directly impacted [2], especially marginalized communities [1] who disproportionately bear the impact of flawed technology designs [4]. Beyond responsible system design, many scholars and practitioners advocate for public participation in AI design and governance to support community self-determination and decision-making power [2]. In the field of responsible AI—the study of fairness, accountability, and transparency of AI systems—various participatory methods have been applied to the development of machine learning (ML), including integrating domain experts [5], participatory problem formulation to address impacted people’s real needs [6], collaborating with activists for collecting representative data [7], community-driven data labeling [8], crowdsourced polling for model performance [9], red-teaming for identifying vulnerabilities [10], including advocacy groups in creating resources for accountability [11], and activism to influence research institutions [12].

Despite the broader momentum in this direction, previous work has acknowledged challenges to realizing these goals in practice. Participatory methods can be extractive—creating value for powerful actors like companies but without reciprocal benefit to communities and workers involved [13,14]. Researchers in technology firms and academia have also faced structural challenges in integrating participatory methods into the constraints imposed by product team timelines [15–18] against the longer time horizon of community-based participatory methods [7,19,20]. Despite the challenge of adapting participatory methods within real-world constraints, it is within rich organizational context that the benefits of participation can be more holistically assessed.

In this paper, we review the value of participatory methods in ML development and overall project goals. Specifically, we focus on the application of participatory methods in developing ML models for Human Immunodeficiency Virus (HIV) care. First, we provide an overview of the long-standing use of participatory approaches in HIV care and their recent integration into participatory ML for developing HIV care models. We include a real-world case study of the HEROIC-PrEP project, short for Health Record Optimization for Identifying Candidates for Pre-Exposure Prophylaxis (referred to as HEROIC), which aims to develop AI systems for identifying individuals at risk of HIV and enhancing pre-exposure prophylaxis (PrEP) uptake to improve HIV prevention. We then draw key lessons and insights from this process, offering valuable perspectives for the participatory ML community. The paper provides guidance on shaping participatory ML projects both within and beyond healthcare and presents lessons that can advance the field of participatory ML on a structural level.

## 2 Use of participatory methods for HIV care

Healthcare organizations are uniquely poised to support a community-based participatory approach. First, their incentive structures are aligned with promoting patient safety, quality of care, and wellness outcomes, especially after the Affordable Care Act, colloquially known as Obamacare, increased accountability for patient outcomes and imposed financial penalties for low-value services. Second, medical training emphasizes the importance of addressing health inequities at every level—from individual patient outcomes to economic and geographic disparities in access to care. Third, healthcare services remain highly local, relying on direct person-to-person relationships with patients. Over the 41-year history of the HIV epidemic, community activism has laid the foundation for participatory research and strongly shaped the scientific and public health agenda.

### 2.1 Evolution of participation: From patient-led to professional activism

HIV has long affected marginalized populations, requiring people with HIV and the clinicians to organize and demand accessible treatments [21]. People with HIV in the US were actively neglected by government institutions at first. Ronald Reagan infamously did not speak publicly about HIV until 1985—more than four years after the epidemic erupted in major urban areas across the country. People with HIV organized and became lay experts in the science of clinical trial design, the Food and Drug Administration’s drug approval process and strategy, and the pathways of HIV research investment at the NIH and in the pharmaceutical industry [22]. These patient-led organizations won seats at the table at the NIH and pharmaceutical companies, contributing their voice to the design of HIV research trials, the choice of which drugs to pursue, and the development of treatment guidelines for the medical establishment [22].

Once effective HIV treatments emerged, clinicians mobilized to highlight the needs of people living with HIV in the Global South to academic and international institutions. In 2000, HIV overtook tuberculosis to become the leading infectious cause of death among adults and 90% of deaths were in poor countries [23]. In 2001, Dr. Paul Farmer, a medical anthropologist, HIV clinician, and co-founder of Partners in Health (PIH), criticized scientific meetings for neglecting the challenge of providing Antiretrovial Therapy (ART) to those in poverty with HIV. He noted that although 90% of potential beneficiaries are in poor countries and access to treatment is a central concern for affected communities and activists, it is rarely discussed at scientific congresses [23]. PIH successfully demonstrated HIV treatment effectiveness in rural Haiti and played a pivotal role in advocating for HIV treatment funding in the Global South [24].

To ensure HIV treatments remain accessible to those who need, people with HIV and their clinicians jointly fought against technology firms controlling HIV treatment access. In 1995, the World Trade Organization (WTO) mandated patent grants in the Global South [25]. Pharmaceutical companies sued governments to restrict local production of generic medications. Countries like India, Brazil, and Thailand, that had successfully treated HIV patients with locally produced pills, began limiting access to treatment. In response, patients and clinicians advocated for change, leading the WTO to agree in November 2001 that trade laws should support WTO Members’ public health and access to medicines [26].

The ongoing monitoring of HIV trends and outbreaks involved the active participation of people with HIV and their clinicians in a surveillance effort. Traditional HIV surveillance has primarily functioned as a public health tool for assessing the state of the epidemic in a given population [27]. For example, the U.S. Centers for Disease Control and Prevention (CDC) compiles HIV surveillance reports that track diagnoses by geographic region, age, race, sex, and transmission category [28,29]. In contrast, participatory surveillance actively involves people with HIV and their communities in the collection and interpretation of health data, providing unique information that is not available through traditional surveillance. For example, Brooks et. al [30] consulted with community representatives in three rural South African villages and identified a range of social and health system factors driving HIV mortality. Swain et. al [31] consulted with HIV care providers and consumers of HIV care to create region-specific HIV care data to inform care strategies in New York State. Advancements in technology facilitated participatory surveillance with enhanced accuracy, sensitivity, timeliness, cost-effectiveness, flexibility, and scalability [32]. Participatory surveillance not only supports timely detection and response to emerging trends but also centers the perspectives of those most affected by HIV in shaping public health action.

### 2.2 Community-based participatory research

Patient and professional activism in HIV care has been crucial in advancing community-based participatory research (CBPR), which fosters strong relationships with marginalized communities and ensures their active participation through its foundational principles, including collaborative partnerships, co-learning, community relevance, and power sharing [33]. CBPR moves beyond data contribution by fostering co-ownership of both the research process and the solutions it generates. While participatory surveillance centers on understanding disease trends through community-informed data, CBPR emphasizes co-development of interventions that directly respond to community-identified needs. In HIV care, CBPR enables communities to shape culturally sensitive, context-specific interventions that reflect their needs and perspectives.

For example, Hullur et. al [34] partnered with community representatives in rural South Africa to examine the causes, treatments, and related issues surrounding deaths due to HIV/AIDS and violent assault. They generated preliminary insights for co-developing remedial strategies. Brown et. al [35] engaged people living with HIV to co-create care recommendations tailored to the needs of aging individuals in the community.

These studies underscore how CBPR promotes more meaningful, actionable research by integrating local knowledge and priorities into the development of HIV care strategies. By adopting CBPR principles, HIV care and prevention programs can be better equipped to meet the diverse needs of affected communities and improve long-term health outcomes.

### 2.3 Participation in ML development

With the emergence of AI technologies in healthcare, numerous ML models have been developed in the last six years for HIV prevention. The participatory model continues to play an important role in the development of these ML models. For example, in 2018, Feller et. al developed an algorithm incorporating unstructured text in clinical notes among NewYork-Presbyterian patients in New York City [36]. In 2019, Marcus et. al [37] developed an incident HIV prediction tool among Kaiser Permanente patients in Northern California. Krakower et. al [38] developed an incident HIV prediction tool among Atrius Health patients in Massachusetts, and Ahlstrom et. al [39] leveraged nationwide registry data in Denmark to predict HIV. In 2022, the first AI system was developed and validated in the US South among Duke Health patients in North Carolina [40]. In these studies, expert clinicians were involved in the development and validation of these algorithms, including activities related to feature selection, outcome definition, case adjudication, and model validation. But these studies reported minimal patient and community engagement to inform the development of any of these algorithms. Furthermore, these algorithms were all developed under research protocols approved by institutional review boards with waivers of written informed consent for use of patient data.

In parallel to model development efforts, researchers have sought to understand the concerns and perspectives of primary care providers (PCPs) and patients in relation to AI systems. Gilkey et al. [41] recruited 31 PCPs and 32 Men who have Sex with Men (MSM) participants in Boston and found that almost all MSM participants “questioned the ability of a tool used at a single point in time to characterize the complex behavior of individuals or generate an accurate estimate of HIV risk.” These men noted that sexual behavior changes dramatically with relationship status, financial circumstances, substance use, and mental health. In addition, the men felt their risk was highly intertwined with their partners’ behavior, which could be difficult to discern. Mootz et. al [42] conducted focus group discussions with 57 MSM participants in New York City focused on topics related to acceptability of AI system use for HIV prediction. In that context, participants thought an AI system could benefit others, but not themselves, asserting their confidence in understanding their own risk of acquiring HIV [42]. Focus group participants, who were 37% Black and 26% Latinx, thought the AI system would be most useful for marginalized populations, such as individuals with housing instability or substance disorder. The MSM in the New York City focus groups emphasized the importance of information about HIV risk being communicated by an empathetic clinician who values the relationship with the patient. The authors summarized that “feeling safe, accepted, and having an established relationship with a licensed and regulated health provider was important to participants” [42]. Van den Berg et. al [43] conducted focus groups with PCPs in Massachusetts and found that PCPs generally believed AI systems would be valuable for identifying PrEP candidates that could otherwise be missed.

Only one study specifically examined PCP perceptions of an AI system in the U.S. South, with the goal of incorporating feedback from clinicians to refine implementation strategies [44]. Potential barriers to AI system use by PCPs included: concerns about incremental workload resulting from AI system implementation and the lack of support staff to help with paperwork for financial assistance for PrEP; clinician biases in assessing HIV risk and insufficient knowledge to discuss HIV risk with patients; and navigating stigma surrounding HIV risk [44]. Overall, PCPs were enthusiastic for an AI system to improve PrEP uptake. Focus group participants also encouraged the research team to broadly engage more stakeholders as part of the AI system roll-out “by having educational materials for the general public, potential PrEP clients, and clients newly initiating PrEP” [44]. Broad participation was felt to be critical to the success of AI system implementation.

Lastly, a recent study described findings from a prospective pilot of an HIV prediction AI system in three community health centers [45]. In that study, the research team developed a novel algorithm tailored to the implementation context and developed a suite of tools for PCPs to discuss HIV risk with patients and prescribe PrEP. As part of the AI system, the team also included support functionality to assist with laboratory test ordering, vaccine administration, diagnosis codes for billing, and templates for documentation. Following discussions with providers in which the importance of prioritizing their traditional mental models of HIV risk became apparent, the research team notably modified the AI system “to flag both patients who were above the risk-score threshold from our model and those who matched providers’ traditional criteria” [45]. While these changes reduced model specificity, the research team “believed that integrating providers’ traditional decision-making criteria would enhance their trust in the tool, which could improve usage and affect PrEP provision” [45]. These findings emphasize the critical role of participation and the importance of distinguishing effectiveness of an AI solution from technical performance of a model. Participatory ML can and should lead to changes in AI system design with the goal of improving solution performance.

### 2.4 Participation in HEROIC: Case study

**Fig 1:**
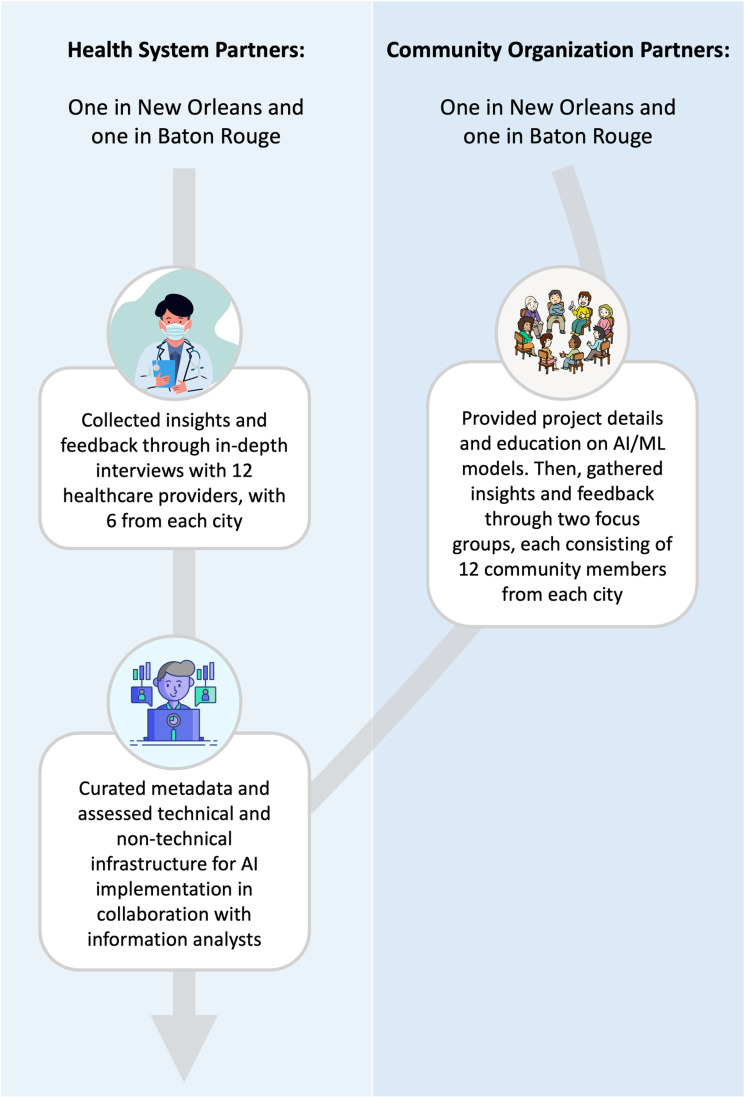
Overview of HEROIC illustrating the engagement of health system partners and community organization partners across key project activities.

HEROIC (Fig 1) was launched in response to the End the HIV Epidemic (EHE) initiative, which mandates participatory methods and active community engagement [46]. HEROIC aims to improve HIV prevention by addressing barriers to PrEP uptake in Louisiana, a state with one of the highest HIV incident rates and lowest PrEP uptake in the U.S. It leverages emerging technologies [47] with a focus on developing ML models to promote PrEP uptake in Orleans Parish and East Baton Rouge Parish. By providing data-informed tools for HIV risk assessment, the project emphasizes objective AI-driven outputs, reducing bias associated with subjective provider assessments and ultimately increasing PrEP adoption.

In 2022, the team secured a 5-year grant from the National Institutes of Health (NIH). Led by two infectious disease physicians at academic medical centers Duke University and Louisiana State University (LSU), HEROIC leverages Duke University’s AI expertise and LSU’s extensive network of affiliated hospitals and clinics across Louisiana. The network includes a large private health system in New Orleans and a large Catholic health system in Baton Rouge, both providing safety net services to low income and marginalized populations. The team partnered with these health systems for the model’s implementation. HEROIC also collaborates with two community organizations—one in New Orleans and one in Baton Rouge—to reach marginalized patients not longitudinally engaged in healthcare [48,49] and implement tools tailored to these regions.

HEROIC recognized the importance of incorporating perspectives from both community members and providers early in the model development process. The team convened two focus groups with 12 members from each community organization to gather input on: 1) local HIV epidemiology, 2) feedback on the baseline HIV risk prediction model, 3) new context-specific features to add into the model, and 4) implementation strategies. To foster mutual understanding, prior to each session, the team shared details about the project, including the study’s motivation, baseline model features, and key questions to consider leading up to the focus group. The team provided background on AI/ML models, explaining: 1) what are AI/ML predictive models; 2) how do they function in the context of healthcare; 3) what are the data sources of these models; and 4) how these models can help clinicians better identify persons who could benefit from PrEP. Concurrently, in-depth interviews were conducted with six clinicians from New Orleans and six clinicians from Baton Rouge, including hospitalists, primary care providers, emergency department providers, and infectious diseases specialists. These interviews covered the same topics as the focus groups.

Focus groups with community members yielded feedback and concerns regarding workflow design and implementation. Participants highlighted the limits of a “risk-based” approach that condenses risk to a single numerical value. Instead, they advocated for a warmer interpersonal encounter where health workers share prevention strategies tailored to individual patient’s needs. One participant described how an encounter could evolve from a person-to-person learning experience, with the ML system output being framed within the context of a meaningful, patient-centered conversation rather than as a data point within an information system. Participants also emphasized the importance of delivering model output results through a “trusted voice.” They advocated for the inclusion of clear, actionable information, such as an itemization of the risk factors (i.e., model features) that contributed most to an elevated risk for HIV acquisition. Additionally, participants raised concerns about how model results would be communicated during moments of acute health-related crises, such as in an Emergency Department (ED) setting, where sensitive information may require careful, empathetic delivery.

Interviews with providers also revealed feedback and concerns about workflow design and implementation. Participants emphasized that the intervention should aim to support frontline staff without increasing their workload. A particular concern was the potential for the AI system to contribute to alarm fatigue, especially in an ED setting. Participants also highlighted that frontline staff—such as medical assistants, nurses, and nursing assistants—who often have the most trusted relationships with patients and “spend more time with patients than anyone,” should be the primary users of the AI system, rather than doctors.

The team is currently in the model development phase of the HEROIC project. They have curated metadata for model development and assessed the technical infrastructure needed for integration. To standardize healthcare data through the creation and maintenance of data ‘groupers,’ [50], they collaborated with informatics analysts and clinicians from both health systems. Additionally, the team guided the health systems in identifying existing technical and non-technical components required for AI integration to ensure an optimal path for implementation. As the HEROIC project continues, it will take several years to fully realize the benefits of participatory ML for HIV prevention. However, the structure of the project provides valuable insights for others seeking to integrate community participation into ML and AI research.

## 3 Key lessons to inform future participatory ML projects

After examining prior research on participation in HIV care, along with insights from a case study, we identified key takeaways that can inform others undertaking participatory ML projects. These lessons are intended to be generalizable and provide actionable guidance for integrating participatory approaches in ML development.

### 3.1 Participation must account for the legacies of institutional harm

ML development relies on personal data, making it essential to ensure representation from all populations, including those historically marginalized. Institutional harm and neglect have led to data gaps and distrust, reinforcing inequities in healthcare and beyond. Recognizing these harms is a crucial first step in identifying excluded communities and ensuring their meaningful participation in AI development. The history of participation in HIV care highlights the importance of engaging marginalized populations in sharing research and securing resources. Similarly, participatory ML must actively involve these communities—not just as data contributors but as decision-makers—to ensure AI systems address real-world needs rather than perpetuate bias. To foster participation, institutions must rebuild trust as an initial step towards inviting participation. In practice, it involves creating spaces for open dialogue that acknowledge past harms, listen to concerns, including anger, and demonstrate a willingness to adjust AI systems based on community input. Without this foundation, efforts to develop equitable ML risk exacerbating existing exclusions.

### 3.2 Participation builds on prior relationships and deepens them

Participatory work is made possible through strong collaborative relationships between researchers and community partners. It is an especially critical foundation when working on stigmatized health issues such as HIV. For example, the relationships between the HEROIC project team and community partners were developed through years of prior work, long before the current project. At the personal level, a key organizer of the community partner had collaborated with the PI at LSU for four years on HIV testing and PrEP advocacy projects. At the organizational levels, both community organizations were already affiliated with LSU through their roles as community health implementation partners. We find that researchers hoping to undertake participatory ML should start by fostering long-term institutional partnerships with community-based organizations, rather than short-term engagements.

### 3.3 Participation means hearing what people say and being willing to change your product roadmap

Meaningful participation requires prioritizing partner input and using it to guide the project’s direction. The project team should be prepared to revise the strategy and implementation plan based on input. In case of the HEROIC project, focus groups emphasized the importance of patient-centered communication, a trusted voice in delivering model results, and the inclusion of diverse prevention strategies. Interviews highlighted trustworthy patient interactions and raised the concerns about alarm fatigue. These insights prompted the project team to revise the workflow design and implementation plan. ML projects using participatory methods should allocate time to revise or re-imagine a project plan; longer timelines are necessary to demonstrate that a project team values the scientific insights of community partners and to build relationships of mutual respect. For the HEROIC project, a 5-year grant timeline supports this approach.

### 3.4 Participation means not being able to do everything people ask for and being willing to go upstream

In practice, a project team cannot address every suggestion from community partners. For example, in the HEROIC project, many data elements recommended by focus groups, such as sexual behaviors, housing stability, and other markers of socioeconomic status, were not well-documented in electronic health record (EHR). This finding highlights that community participation occurred too far “downstream,” relying on existing data and systems without involving community-based organizations in the initial design of the health information system or data identification process.

To address such challenges, participatory ML projects should encompass “upstream” data collection, curation, and storage, extending far beyond the AI system itself to include the definitions of key variables and the in-person processes for data collection. When working with existing data, project teams should clearly communicate what can and cannot be achieved with the current dataset and what additional steps are needed to fully adopt their input in the future.

Our team prepared focus group participants by briefing them on the available EHR data and its limitations, which helped facilitate a more robust and productive discussion. Even with these constraints, participants identified key variables, such as the number of pregnancies or the risk behavior of their sexual partner(s), which offered valuable insights into how social history might relate to risk profiles. While these variables were not feasible with the data available, they highlight that important factors understood by community members are often missing or inadequately captured in the EHR.

### 3.5 Partners will require technical support, which can build capacity long term

Project teams must support partner organizations with personnel, time, technical, and financial resources to aid their engagement in ML development. This investment helps build capacity within community-based organizations and healthcare delivery organizations for future data and technology projects. Capacity building occurs at both organizational and individual levels. For example, HEROIC fostered innovation in one implementation site that had created a local ‘grouping’ tool that surpassed commercial solutions on the market. The HEROIC team also educated community members on AI, EHR, medical data, and data usage, which helped them to be more active participants not only in project decision-making, but in their own care.

## 4 Key lessons to advance the future of participatory ML field

Building on the insights identified earlier, we further elaborated key takeaways for supporting participatory ML the field more broadly.

### 4.1 Establishing funding or hard law requirements promotes participation

Establishing clear requirements for public participation offers significant benefits. Michele Gilman [4,51] argues for the importance of “hard law requirements,” such as the 1970 U.S. National Environmental Policy Act, which mandated community consultation for major development projects that would change the local ecosystem. Similarly, the U.S. CDC required robust public engagement with communities most severely impacted by HIV/AIDS. The NIH End the Epidemic Program emphasizes collaborating with local communities and health system partners to develop tailored, local solutions rather than a “one size fits all” approach. The DAIDS Clinical Trials Network, funded by the National Institute of Allergy and Infectious Diseases (NIAID) integrates stakeholder input through Community Advisory Boards (CABs) at both local and national levels. Composed of community members affected by HIV/AIDS, these CABs ensure meaningful participation throughout the research process, from study design to implementation and dissemination. By embedding community input into decision-making, the DAIDS network serves as a model for effective participatory approaches applicable to AI and ML research.

### 4.2 Participation is about scaling processes rather than scaling technologies

In the case of HIV prevention, a “one size fits all” approach would create a risk detection model that is scaled across geographical and institutional contexts. Ignoring local context would undermine the effectiveness of interventions, given the significant variation across settings in the social inequalities that shape the HIV epidemic, as highlighted by participatory surveillance. Where participatory methods are used to create interventions responsive to local social context, that intervention cannot be exported to another setting. However, processes and best practices used to develop the intervention through local participation can be refined and adapted for use across settings. For example, participatory surveillance methods can support the evaluation of data quality and the identification of contextually relevant features, critical for valid algorithm development. Importantly, this is not to say that participation cannot scale—rather, it is the participatory development process that must scale, rather than the technology solution itself.

### 4.3 Meaningful participation requires a shift in power and leadership support

As practitioners seek to apply participatory methods to the development of ML systems, some approaches merely consult rather than genuinely collaborate, missing the original values and motivation of the participatory tradition to shifting power. Delgado et al. [2] refer to these efforts as “consultation” rather than meaningful collaboration and community-ownership. Sherry Arnstein’s 1969 work [52] emphasize that meaningful participation involves a shift in decision-making power, requiring collaboration of those already in authority.

For institutions to embrace participatory methods, participation must be driven by individuals in positions of authority. In HEROIC, the co-PIs were committed to making changes to the product roadmap based on partner feedback. Without such leadership, marginalized groups must advocate for change, gradually moving from the least empowered to the most empowered stakeholders. This step-wise advancement of participation in HIV advocacy carries important lessons for technology corporations. Just as clinicians became key partners in advocacy for people living with HIV, technology professionals must champion marginalized groups to drive meaningful participation and change institutional practices.

## 5 Conclusion

In this paper, we review the intersection of participatory practices in HIV care with the emerging focus on responsible AI. Our first contribution is to connect the long-standing tradition of participatory approaches in HIV medicine with the recent scholarly record in participatory methods in AI research. Through this effort, we aim to enhance the understanding of how participatory ML practices can be applied to ensure that AI systems are developed with greater sensitivity to diverse needs and contexts.

Our second contribution is to provide a case study of a participatory ML project aimed at developing a tool to identify candidates for PrEP. The case study illustrates the multifaceted nature of participatory ML, including the establishment of collaborative relationships at multiple levels in the local context—between the project team and partner health systems, community-based organizations and their staff, as well as with patients. It also highlights that the project is not limited to eliciting input from participants, but also encompasses robust resource-sharing in metadata curation and capability assessment for system integration. Attention to these multiple levels and forms of participation illuminate what a narrow focus on the technical gains to model performance may miss: that capacity-building with partners is a primary value of participatory ML—not solely model performance gains.

Finally, we offer lessons for future participatory ML projects. Namely, the value of establishing long-term, collaborative relationships. Genuine participation may disrupt pre-existing plans and timelines, making it essential for the project team to be responsive to this input, even if not all proposed changes can be implemented due to upstream system constraints. The “hard” funding requirements that mandates participation, combined with insights from other projects on effective engagement, can empower ML and AI research projects to confront established practice with the truth of needs and realities on the ground.

While our work is grounded in the US context, it carries significant implications for global practices in participatory ML. By thoughtfully adapting our work, we hope to contribute valuable insights for adapting participatory methods across various contexts and ultimately advance the responsible development and application of AI technologies worldwide.
